# Impact of antibiotic choice on immune response and antibiotic resistance development in piglets experimentally infected with *Escherichia coli*

**DOI:** 10.3389/fcimb.2025.1627782

**Published:** 2025-09-09

**Authors:** Leon Cantas, Christopher G. Fenton, Emese Bato, Ruth H. Paulssen, Henning Sørum

**Affiliations:** ^1^ Department of Food Safety and Infection Biology, Norwegian School of Veterinary Science, Oslo, Norway; ^2^ Norwegian Private Veterinary Services, PrivateVet.no, Hammerfest, Norway; ^3^ Department of Clinical Medicine, Clinical Bioinformatics Research Group, Faculty of Health Sciences, UiT- The Arctic University of Norway, Tromsø, Norway; ^4^ Xenovea Ltd., Szeged, Hungary; ^5^ Department of Paraclinical Sciences, Faculty of Veterinary Medicine, Norwegian University of Life Sciences, Ås, Norway

**Keywords:** ETEC, piglet, R-plasmid, innate immunity, gut microbiota, antibiotic resistance

## Abstract

The rapid mobility of antibiotic resistance genes (ARGs) represents a growing global One Health concern. However, further *in vivo* studies are needed to better understand ARG dissemination in actual clinical settings. To this end, a piglet model of enteric colibacillosis with the causative bacterium carrying an R-plasmid, was used to track the expression of genes involved in the bacterial SOS response, plasmid transfer, and porcine immune responses under both effective and ineffective antibiotic treatments. Analysis of gut samples showed a significant reduction (*p* < 0.05) in the expression of R-plasmid transfer genes in groups receiving effective enrofloxacin, with or without probiotics or meloxicam. Conversely, ineffective tetracycline and sub-inhibitory enrofloxacin resulted in a significant increase (*p* < 0.05) in the expression of bacterial SOS response and R-plasmid transfer genes. Inflammatory gene expression was upregulated in the groups receiving ineffective antimicrobial treatment, whereas anti-inflammatory cytokines exhibited the opposite trend in effectively treated piglets. These findings highlight the importance of selecting the correct antibiotic and administering it at an effective dosage. The improper use of antibiotics or their administration at subinhibitory concentrations can result in high mortality/morbidity rates and accelerate the spread of ARGs.

## Graphical Abstract

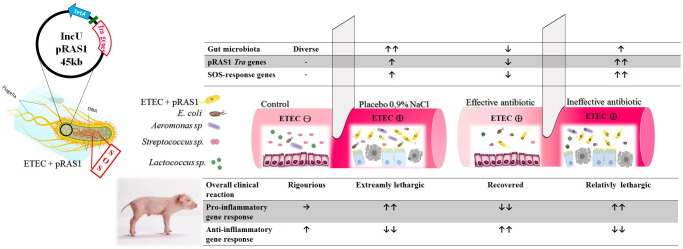

## 1 Introduction

The development and spread of multidrug-resistant bacteria poses a severe threat to human and animal health as well as to the environment (Velazquez-Meza et al., 2022). The use of antibiotics inevitably results in the appearance of novel strains of resistant pathogenic bacteria. Until recently, the development of novel antibiotics offered a means of combating these new resistant strains (Terreni et al., 2021). However, it is increasingly evident that this approach is not medically sustainable over the long term because bacterial resistance is evolving more rapidly than the discovery of new antibiotics (Aminov, 2010; Ventola, 2015).

The horizontal transfer of antimicrobial resistance genes (ARGs) through conjugation, transformation, and transduction represents a major adaptive survival strategy for bacteria, and is a major contributor to the development of multidrug resistance (MDR) among pathogenic bacteria (Baharoglu et al., 2010; Vrancianu et al., 2020). The conjugative transfer of antibiotic resistance genes (ARG) carried by resistance plasmids (R-plasmids) is the most prevalent route for MDR development in a range of bacterial genera and species (Leungtongkam et al., 2018). Most ARG transfer-related experiments to date have been conducted *in vitro*, which may not accurately reflect the real-world dynamics in living hosts (Porse et al., 2017; Tao et al., 2022).

In this study, we used an IncU plasmid, pRAS1, which displays a high frequency of transfer among diverse groups of gram-negative bacteria. pRAS1 was originally isolated from an opportunistic fish pathogen, *Aeromonas salmonicida*, in Norway, but has since been isolated from other microbial species in different habitats (Sørum et al., 2003). This plasmid was found to harbor identical genes to those present in plasmids isolated from human pathogenic *Escherichia coli* causing urinary tract infections in Germany (Tschäpe et al., 1984).

The gap in our understanding of ARG dissemination between *in vitro* and *in vivo* settings prompted us to establish a mammalian infection-treatment model, aiming to mimic field-like conditions. To demonstrate cross-species transmission, pRAS1 was experimentally transferred by conjugation to one of the major causative agents of neonatal diarrhea in young piglets, enterotoxigenic *E. coli* (ETEC). The course of infection and clinical status of the piglets were monitored *via* regular examinations and the culturing of fecal bacteria. Intestinal samples were collected for conventional bacterial cultivation and histopathologic analysis. Simultaneously, changes in the expression levels of genes involved in the bacterial SOS response and R-plasmid transfer and the contents of pro-/anti-inflammatory cytokines in response to effective and ineffective antimicrobial treatment were investigated in the gut samples. The results indicate that antibiotic use should primarily be employed when the causative bacterium has high susceptibility to the chosen antibiotic. Additionally, it is important that the infected patient is able to mount a strong immune response.

## 2 Methods

### 2.1 Bacterial strains and growth conditions

Atypical *Aeromonas salmonicida* 718 (NVI 2402/89), originally isolated from the head kidney of diseased Atlantic salmon in 1989 and harboring a 25-MDa conjugative IncU plasmid, pRAS1, mediating resistance to oxytetracycline, trimethoprim, and sulfadiazine, was used as the donor strain (Sørum et al., 2003). ETEC (serotype O149, F4) (NVH), initially isolated from the kidney of a piglet with severe diarrhea, served as the recipient strain. Both strains were incubated at 22°C on 5% cattle blood agar (Blood Agar Base No. 2, BD Difco) for 48 h.

### 2.2 *In vitro* conjugation

Donor *A. salmonicida* 718 (carrying plasmid pRAS1) and recipient *E. coli* O149 (NVH) strains were grown overnight in Luria Broth (LB) with shaking at room temperature. These cultures were diluted in LB to approximately 1 × 10^8^ CFU/mL. Donor and recipient cultures (100 µL each) were mixed and transferred to a sterile 0.45-µm filter (Millipore) before being placed on an LB agar plate and incubated for 24 h at 22°C. The resultant colonies were suspended by vortexing the filter in 1 mL of LB, pelleted, and resuspended in 100 µL of the same medium. For selection of transconjugants, serial dilutions were spread onto selective Luria agar (LA) plates supplemented with tetracycline (10 µg/mL), trimethoprim (10 µg/mL), and sulphadiazine (200 µg/mL), followed by incubation at 37°C for 24 h. In parallel, the total number of recipients was estimated on LA after 24 h of incubation at 37°C, a non-permissible temperature for the donor strain. Conjugal transfer frequencies were calculated by dividing the number of transconjugants by the number of *E. coli* recipients. The frequency of pRAS1 transfer was found to be 3.4 × 10^−3^. pRAS1 transfer was confirmed by plasmid profile analysis and the resistance profiles of the transconjugants.

### 2.3 Animals and treatment units

The piglet experiment was approved by the National Animal Research Committee in accordance with the Norwegian Regulations on Animal Experimentation. Four Landrace × Yorkshire sows, selected for synchronized delivery based on artificial insemination dates, were bought from a commercial farm in southern Norway. The sows were transferred to disinfected farrowing pens (7.2 m^2^, with solid floors) at the Terrestrial Research Animal Unit, Norwegian School of Veterinary Science (NSVS), 3 weeks prior to the expected delivery date in the spring of 2012. Before farrowing, the sows were offered approximately 0.2 kg of hay and 1.5 kg of a commercial pig diet as dry feed twice daily with *ad libitum* access to water.

**Figure 1:**
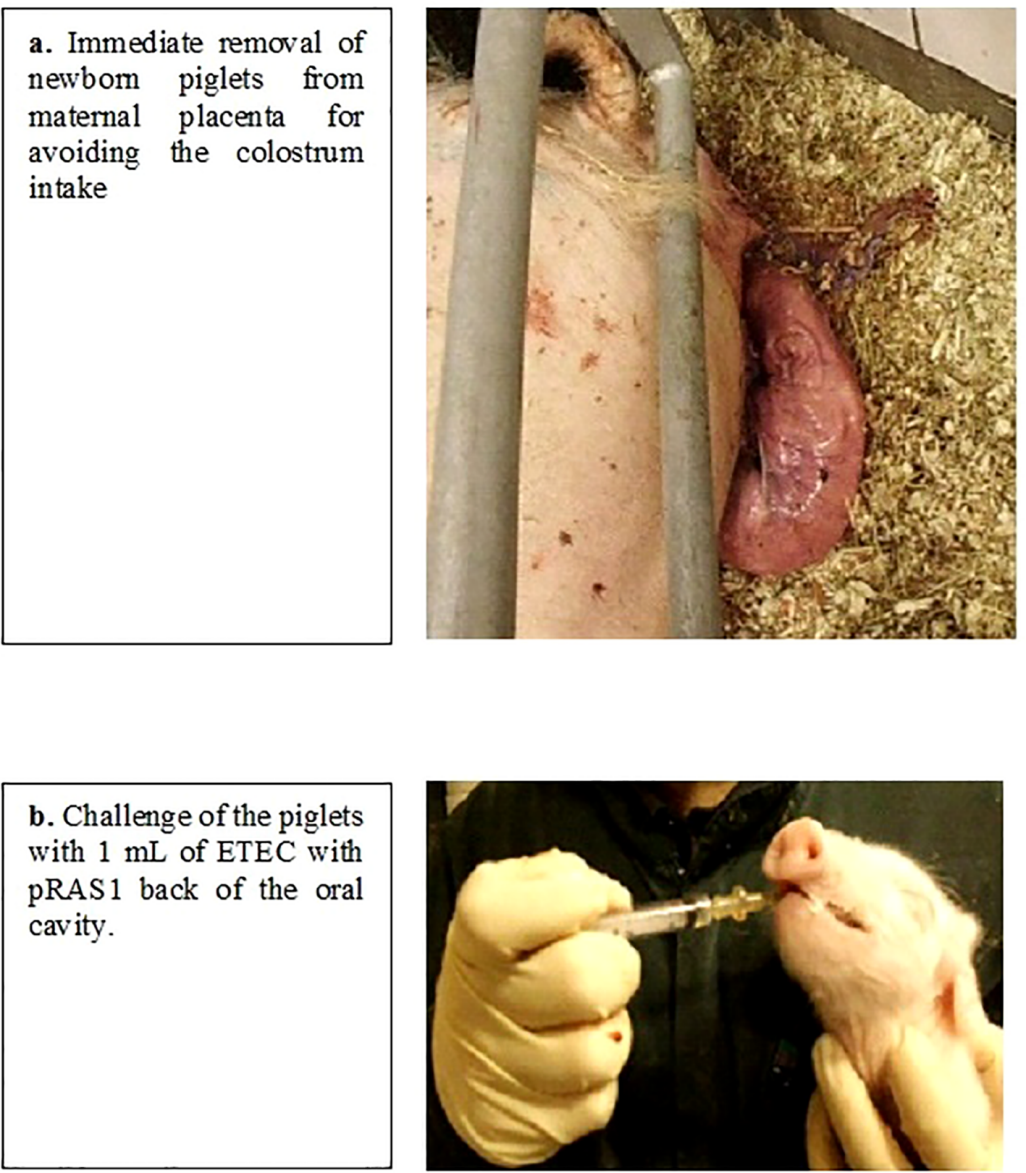
**(a, b)** Initial experiment set-up and infection challenge.

Although delivery was synchronized, farrowing started naturally. The piglets were immediately separated from the sows to avoid colostrum intake (**Figure 1a**) and placed in washed and disinfected pens measuring 1.25 × 1.8 m. From parturition to necropsy, the newborn piglets were fed every 45 min with a commercial milk replacement without blood plasma and salt balance. The commercial milk replacement was available *ad libitum* in low containers placed on the floor of the pens. In addition, they had a heated area with sawdust bedding. Fifty-two newborn piglets were split between different sterilized challenge-treatment units (**Table 1**). There were 25 males and 27 females, with mean weights at birth and necropsy of 1.6 and 1.23 kg for males and 1.5 and 1.24 kg for females, respectively. The following treatments were conventionally used in this study: Terramycin (tetracycline [*Tet*]; Pfizer), Baytril 10% (enrofloxacin [*Enr*]; Bayer), Metacam (meloxicam [*Mx*]; Boehringer Ingelheim Vetmedica GmbH), and Zoolac Propaste (probiotic [*Prob*]; ChemVet Dk A/S).

**Table 1:** Treatment groups, number of pigs in each unit, dosage, and drug administration.

Treatment groups	Impact	Group size	Dosage and administration
No infection, no treatment ^a^	–	5	–
Sterile 0,9% NaCl, H_2_O (Placebo)^b^	IE^c^	5	0.1 mL, IM
Terramycin, Pfizer (Tetracycline)	IE	5	10 mg/kg, IM
< MIC^d^ Baytril 10 %, Bayer (Enrofloxacin)	IE	5	0.03 mg/kg, IM
> MIC Baytril 10 %, Bayer (Enrofloxacin)	E^e^	5	5 mg/kg, IM
Metacam, Boehringer Ingelheim Vetmedica GmbH (Meloxicam) + Placebo	IE	5	0.4 mg/kg, IM
Metacam + > MIC Baytril 10 %	E	5	0.4 mg/kg, IM and 5 mg/kg, IM
Zoolac^®^ Propaste (1 mL, PO, ChemVet, dk A/S)	IE	5	2 mL after infection, and 1 mL every 8 hours, PO
Zoolac^®^ + > MIC Baytril 10 %	E	5	1 mL every 8 hours, PO and 5 mg/kg, IM
Zoolac^®^ + > MIC Baytril 10 % + Metacam	E	4	1 mL every 8 hours, PO + 5 mg/kg, IM and 0.4 mg/kg, IM
Zoolac^®^ + Metacam	IE	3	1 mL every 8 hours, PO and 0.4 mg/kg, IM

^a^Challenged with 1 mL of 0.9% NaCl, PO. ^b^Challenged with 1 mL of a 6-h broth culture of pRAS1 bearing *Escherichia coli* (8 × 10^6^ CFU/mL), PO. ^c^IE = Ineffective, ^d^MIC = Minimal Inhibitory Concentration, ^e^E = Effective.

### 2.4 Challenge procedure and treatments

All piglets received 1 mL of a 6-h broth culture of *E. coli* (8 × 10^6^ CFU/mL) containing pRAS1 at the back of the oral cavity (**Figure 1b**). The bacteria-rich solution was slowly dribbled into the piglets’ throats, thereby triggering the swallowing reflex and avoiding entry of the inoculant into the lungs. The uninfected control group received the same volume of sterile physiological saline. The piglets were observed every hour for one day following exposure. Appetite was evaluated post-challenge *via* oral feeding with 20 mL syringes every 45 min (approximately 25 mL each time). After 6–8 h, all the infected piglets developed signs of illness, namely, a yellow-greenish color intestinal content often accompanied by a transparent, severe, watery mucoid diarrhea. Piglets showing signs of dehydration were administered 0.9% saline by intraperitoneal injection, as needed. Two piglets that became moribund (after 26 h) were euthanized for animal welfare reasons. All other piglets were euthanized at the end of the experimental period, that is, 30–32 h post-partum.

### 2.5 Necropsy and intestinal sampling

All piglets were euthanized by blunt force trauma to the head, resulting in the physical destruction of the skull and brain tissue. The intestinal tract was removed from the abdomen immediately after death. Transversal slices (<0.5 cm) of the jejunum were sampled and immediately fixed in 4% formalin for histopathological evaluation, while parallel tissue samples were immersed in RNAlater (Invitrogen) to preserve both porcine and bacterial RNA. All samples were stored at −80°C for advanced metagenomic and proteomic analyses in the spring of 2023.

### 2.6 Bacteriological culture

Jejunal contents collected from each euthanized piglet were examined for the presence of the challenge pathogen and quantification of the intestinal flora. Specimens were streaked on 5% cattle blood agar [Blood Agar Base No. 2, BD Difco].

### 2.7 Analysis of gene expression

Total RNA was extracted, DNA-free, from archival gut samples (22 out of 56) stored in RNAlater at −80°C from 2012 to 2023, as instructed by the manufacturer (Qiagen, Valencia, CA, USA). The integrity of the isolated RNA was determined using gel electrophoresis, while its concentration and purity were assessed using a NanoDrop ND-1000 (NanoDrop Technologies, Wilmington, DE, USA).

For NGS-based gene expression profiling (GEx), libraries were constructed using the QuantSeq 3′ mRNA-Seq Library Prep Kit FWD for Illumina (Lexogen GmbH, Wien, Austria), following the manufacturer’s protocol. Library concentrations were measured on a Fluostar Omega microplate reader (BMG Labtech) using the Quant-iT 1× dsDNA HS Assay kit (Thermo Fisher Scientific). The fragment size distribution of the libraries was determined by capillary electrophoresis on a LabChip GX Touch HT Nucleic Acid Analyzer using an HT DNA X-Mark Chip and the DNA NGS 3k Assay Kit (Revvity). Pooled libraries were diluted to 650 pM for 82 bp single-read sequencing using a 50-cycle P3 Reagent Kit on a NextSeq 2000 Sequencing System (Illumina, San Diego, CA, USA) according to the manufacturer’s instructions. Advanced proteogenomic technologies offer a fast and reliable quantification of the mRNA produced in any target sequence in a sample (Burgos et al., 2002).

### 2.8 Statistical analysis

Gene count data were analyzed using the R package DESeq2 (Love et al., 2014). DESeq2 was used to normalize raw counts and calculate log2 fold changes in gene expression between responses to effective and non-effective treatments. Genus-level abundance was calculated using the Kraken2/Bracken pipeline (Wood et al., 2019) with standard databases and indexes. Bracken was applied to estimate the fraction of each genus in each sample (Lu et al., 2017). For calculating the statistical significance of the differences in the relative fold change between effective and non-effective responses, *p*-values were calculated using z-scores for each value within the respective probability distribution.

## 3 Results

### 3.1 Clinical pathology of infection with ETEC

**Figure 2:**
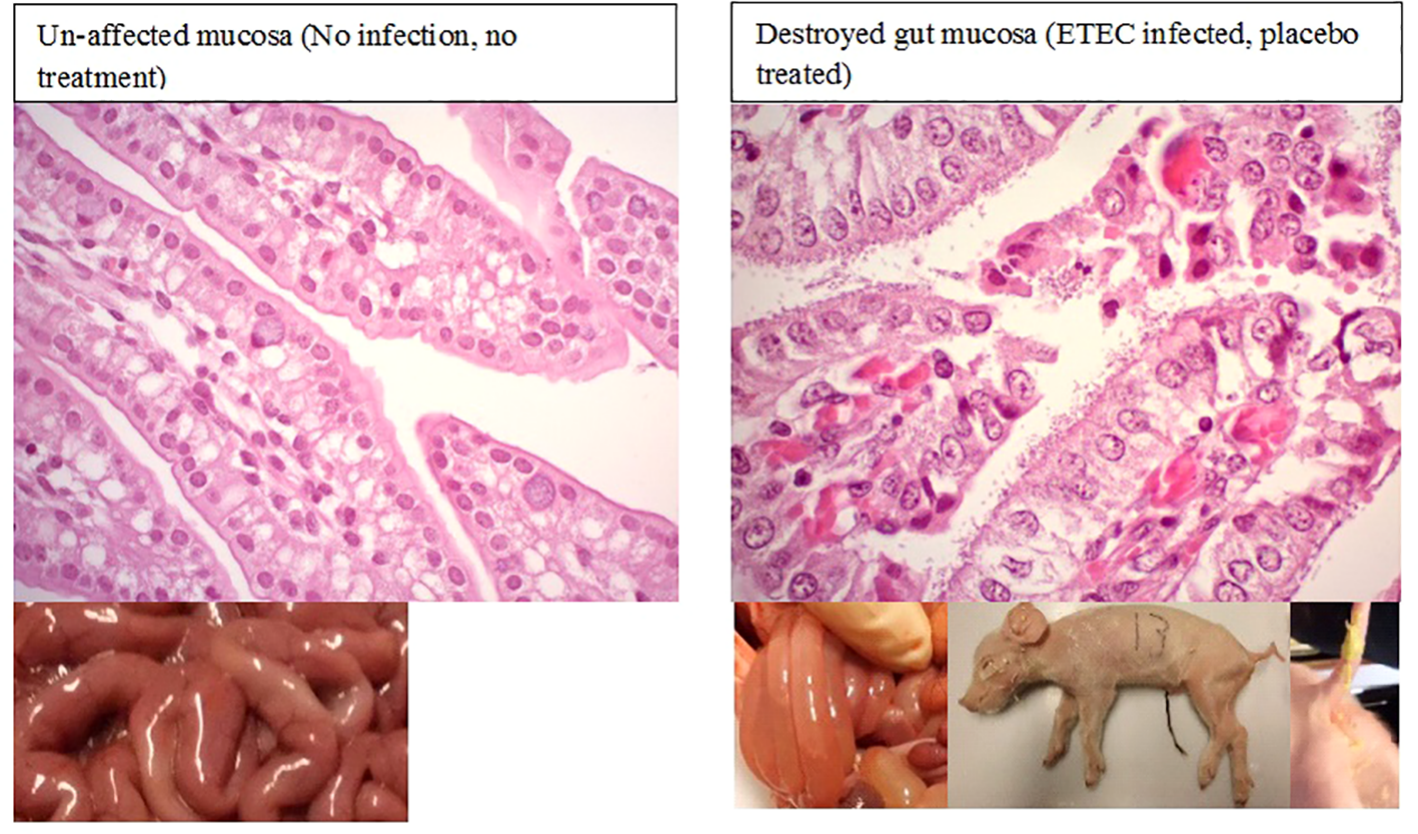
Enterotoxigenic *Escherichia coli-*infected piglets with yellowish mucoid diarrhea. Histopathologically, ineffectively treated piglets exhibited a stronger innate immune response than the control group without infection.

**Figure 3:**
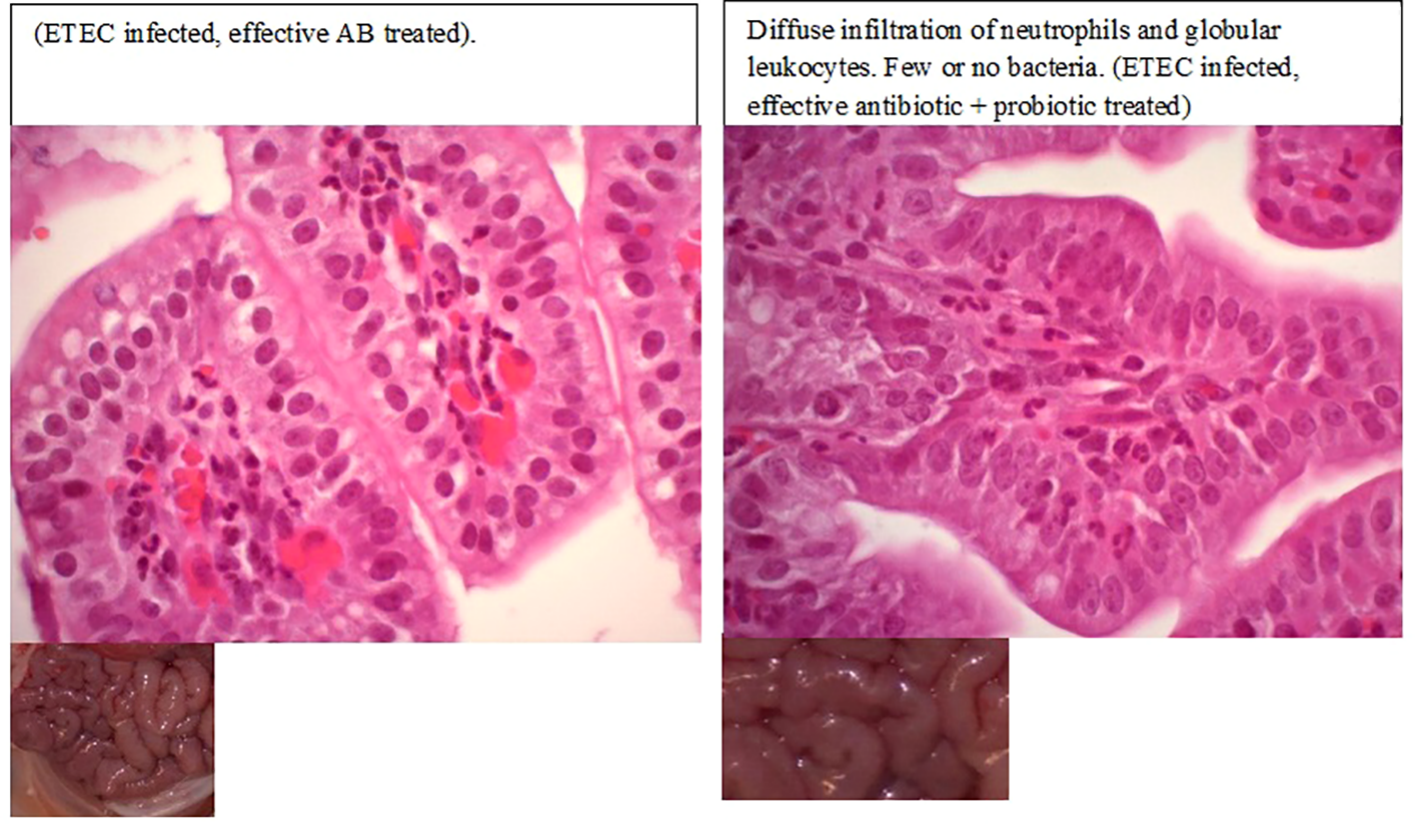
Histopathological changes in piglets with enterotoxigenic *Escherichia coli* infection after effective antibiotic treatment.

All the *E. coli*-inoculated piglets showed loss or reduction of appetite, acute watery or mucoid pale-yellow diarrhea, increased respiratory activity, and decreased vigor within 6–8 h post-challenge (**Figure 2**). In contrast, uninfected piglets remained healthy, displaying normal vigor, appetite, and skin color, and no diarrhea. Macroscopic and histopathological examination revealed the presence of inflammation in the intestinal mucosa in both placebo-treated and ineffective antibiotic-treated piglets following ETEC infection. Evidence of epithelial cell necrosis and degeneration was most apparent in gut sections with a high level of pathogenic bacterial colonization. Meanwhile, all the piglets that were treated only with an effective antibiotic or its combination with a *Prob* or *Mx* survived, and only slight histopathological changes were noted in their gut wall samples (**Figures 2**, **3**).

### 3.2 Changes in the total gut microbiota and SOS response- and R-plasmid transfer-related genes after *E. coli* challenge

Intestinal samples from piglets receiving placebo or ineffective treatments showed moderate to heavy growth of *E. coli* in almost pure culture. In contrast, only a few or no *E. coli* colonies were cultured from piglets receiving effective antibiotic treatment (+/− *Prob* or *Mx*). Bacterial gene copy numbers in the ineffective treatment groups were similar to those in the placebo group. Furthermore, the tetracycline-susceptible microorganisms in the normal gut microbiota were largely eliminated in piglets treated with tetracycline, leaving resistant *E. coli* as the dominant bacteria on the culture plates. A minor reduction in gut microflora was observed in the group receiving a sub-therapeutic concentration of enrofloxacin. Notably, probiotic bacterial colonies were absent in cultures from the intestines of piglets treated with the effective concentration of enrofloxacin.

**Figure 4:**
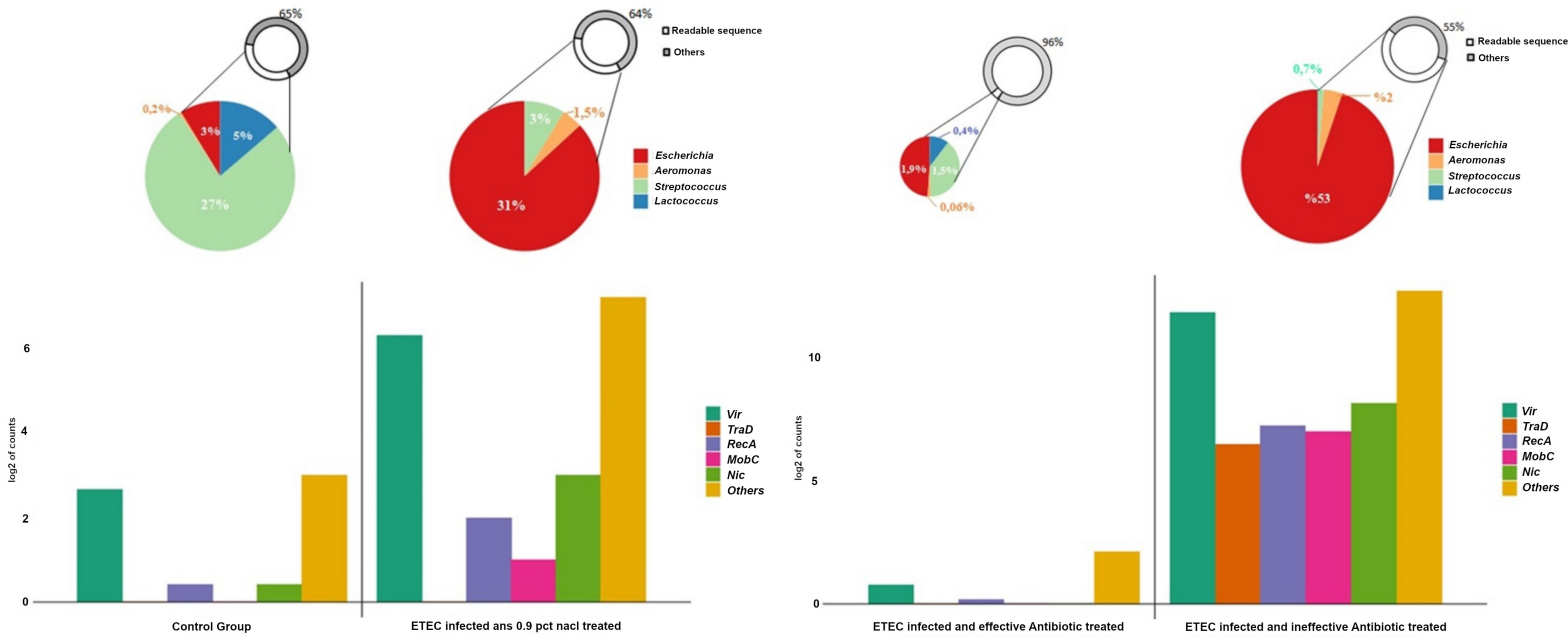
The expression of R-plasmid transfer genes and genus-level bacterial composition. R-plasmid transfer genes (“RecA”, “mobC”, “nic”, “vir”, and “tra”) were isolated from the count matrix. For each gene family, the log2 of the sum of all identified elements is shown (e.g., “vir” represents the log2 sum of 10 “vir” elements). “Others” represents the log2 sum of the remaining DNA metabolism-related genes not included in the specified transfer gene families. Genus-level relative abundance was determined using the Kraken2/Bracken pipeline with standard settings. The percentage of *Escherichia*, *Aeromonas*, *Streptococcus*, and *Lactococcus* was calculated for each treatment group (control, ETEC-infected placebo, ETEC-infected effectively treated, and ETEC-infected ineffectively treated).

The expression of the bacterial SOS response gene *RecA* and the R-plasmid transfer-related genes *mobC, nic, tra*, and *vir*, which mediate the conjugative transfer of pRAS1, was strongly induced in the intestines of infected piglets receiving ineffective treatment (*p* < 0,05). Conversely, effective concentrations of enrofloxacin (+/− *Prob*) were associated with lower mRNA copy numbers of this gene set (**Figure 4**).

### 3.3 The expression of inflammatory and immune response genes in infected piglets

**Figure 5:**
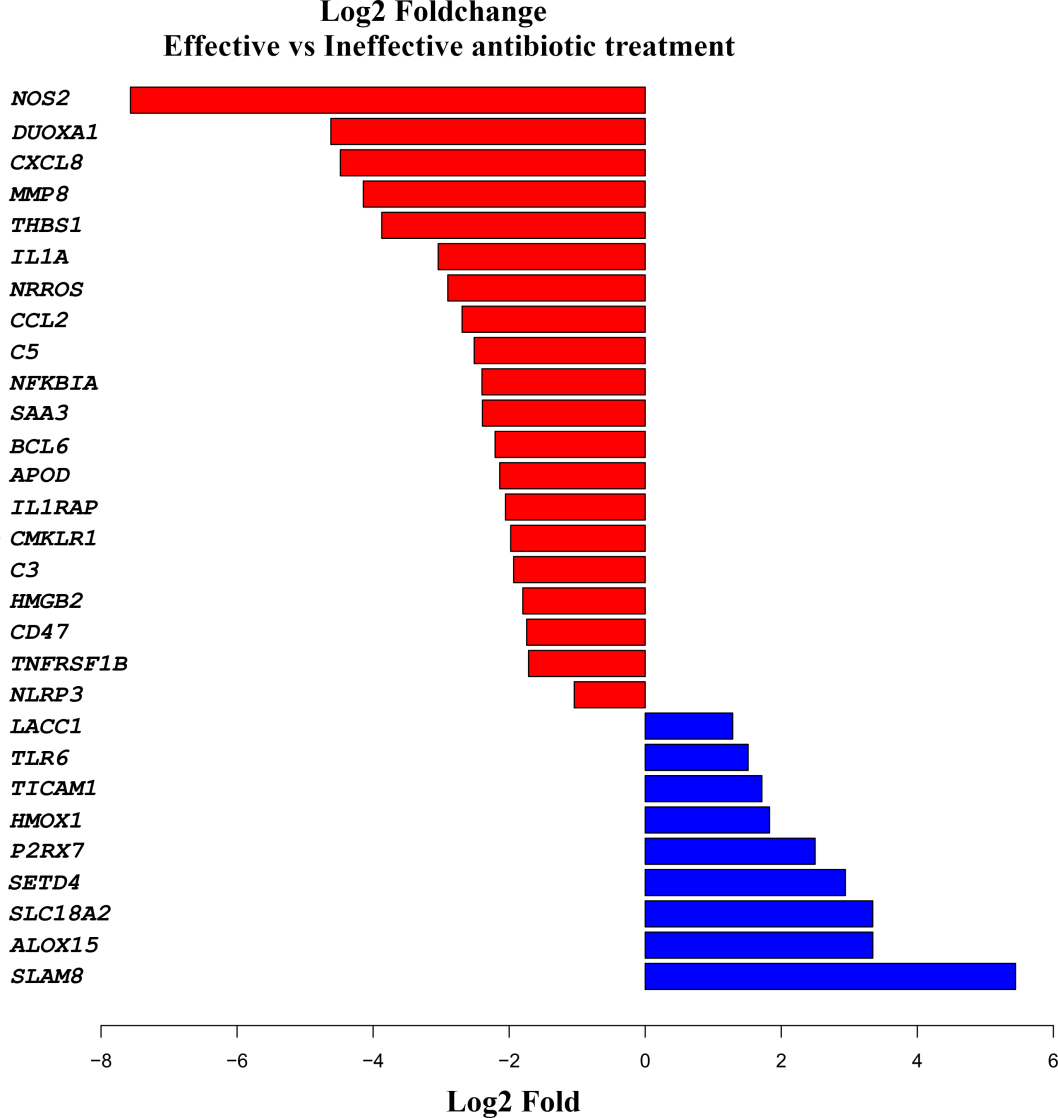
Comparative cytokine gene expression levels in the jejuni of piglets 24 hours post-treatment for enterotoxigenic *Escherichia coli* infection. Genes associated with the “inflammatory response” GO pathway (GO_0006954) were identified using the AnnotationDbi R interface. DESeq2 was used to normalize raw counts and calculate gene log2 fold changes between effective and ineffective treatments. Log2 fold-change values for genes with a *p*-value of <0.1 that are part of the inflammatory response pathway are shown. The plot was generated using the R ggplot2 package (ggplot2: https://ggplot2.tidyverse.org/).

The expression of the selected inflammatory and innate immune response genes in the small intestines of the infected piglets exhibited distinct expression patterns 24 h post-treatment (**Figure 5**; **Supplementary Data Sheet 1**). Infection with *E. coli* resulted in significant inflammatory responses, characterized by the upregulation of the *NOS2*, *DUOXA1*, *DEFB114*, *THBS1*, *CXCL8*, *MMP8*, *NRROS*, *IL1A*, and *C5* genes. Meanwhile, immune-related genes, such as; *SLAMF8*, *SETD4*, *SLC18A2*, *ALOX15*, and *P2RX7* were the most upregulated genes in the intestines of piglets that received effective treatment.

## 4 Discussion

In this study, we established an experimental infection model in piglets using ETEC, which resulted in the anticipated clinical signs of severe diarrhea and lethargy (Krsnik et al., 1999). The re-isolation of the challenge ETEC organism from the intestines, the presence of necrotic pathological changes in the intestinal cell wall, and increased inflammatory gene expression in the ETEC-challenged piglets supported the clinical findings and confirmed establishment of the infection. It has been shown that ETEC produces an array of virulence factors that induce strong inflammatory responses (Smyth et al., 1981). In the present study, *E. coli* infection resulted in notable inflammatory changes, marked by the upregulation of *NOS2*, *DUOXA1*, *DEFB114*, *THBS1*, *CXCL8*, *MMP8*, *NRROS*, *IL1A*, and *C5*. The expression of these genes is a major indicator of an acute inflammatory response in the small intestine, serving to activate the innate immune system to mount a response against the infection (Sahu and Lambris, 2001). Specifically, *NOS2* promotes the production of various radicals, while *DUOXA1* modulates cellular enzyme activities, ultimately contributing to inflammation and tissue damage. Studies (Michelle et al., 2008) have shown that increased levels of *CXCL8* and *MMP8* are associated with neutrophil migration, which is consistent with their increased expression in the small intestines of effectively treated piglets.

The expression of signaling lymphocyte activation molecule family member 8 (*SLAMF8*), encoding a type I glycoprotein found on the surface of immune cells, was markedly increased in the piglets that received effective antibacterial treatment. In addition, a significant increase was observed in the mRNA levels of *SETD4*, which encodes a methyltransferase that plays a role in the regulation of macrophage activity and has been linked to increased production of interleukin-6 (IL-6) and tumor necrosis factor alpha (TNF-α) in response to bacterial LPS stimulation (Zhong et al., 2019). The expression of 15-lipoxygenase (*ALOX15*) and P2X purinoceptor 7 (*P2RX7*) was also significantly increased in the intestinal wall of piglets 24 h after ETEC challenge followed by effective antimicrobial treatment. The proteins encoded by both genes are associated with the regulation of T-cells. ALOX15 has been further linked to inhibition of the immune reaction (Marques et al., 2021) and P2RX7 to cell apoptosis (Welsby et al., 2012).

The expression of solute carrier family 18 (vesicular monoamine transporter), member 2 (SLC18A2) was upregulated in the intestinal tissue of piglets receiving effective antimicrobial treatment. This protein is linked to transmitter transport in the central nervous system (CNS) in humans (Surratt et al., 1993).

The observed significant increase in the expression of selected genes (**Figure 5**; **Supplementary Data Sheet 1**) strongly suggests that the piglets that received effective antimicrobial treatment with enrofloxacin mounted a highly efficient innate immune response. This may indicate that innate immune activity played a crucial role in the efficient control of ETEC-induced diarrhea in piglets treated with effective antibiotics during acute infection.

The gene encoding nitric oxide synthase (*NOS2*) was highly upregulated in piglets with ineffectively controlled ETEC infection. Nitric oxide (NO) is a cellular signaling molecule. NOS2 expression is induced by a combination of LPS and cytokines, and the enzyme is involved in immune activity against both bacteria and viruses (Cinelli et al., 2020). Inducible NOS (iNOS) is only expressed following stimulation by factors such as pro-inflammatory cytokines or LPS (Sharma et al., 2007). Dual oxidase maturation factor 1 (DUOXA1) was also upregulated in effectively treated piglets. This enzyme participates in the regulated production of reactive oxygen species (ROS), which are involved in phagocytic activity and regulate the activity of several other cells (Cross and Jones, 1991). The expression of *DEFB114*, which encodes a beta-defensin, was similarly elevated in effectively treated piglets. DEFB114 is an antimicrobial protein involved in defense against infectious pathogens and possesses multiple features, playing a key role in both innate and adaptive immunity as well as in non-immune processes. The expression of thrombospondin 1 (*THBS1*), which has been linked to the inhibition of neovascularization and tumorigenesis (THBS1 thrombospondin 1 [Homo sapiens (human)] - Gene - NCBI (nih.gov)), was upregulated in piglets receiving ineffective infection treatment. Interleukin 8 (IL-8), encoded by the *CXCL8* gene, was also highly expressed in the ineffectively treated piglets. IL-8 is produced by macrophages and other cell types possessing Toll-like receptors, thus linking it to the innate immune response. When macrophages initially detect an antigen, they secrete IL-8 to recruit other immune cells (Harada et al., 1994).

The *MMP8* gene, encoding matrix metalloprotease 8 (MMP-8), showed elevated expression in piglets receiving ineffective treatment for colibacillosis. MMP-8 breaks down extracellular collagen and has been implicated in the production of IL-6 and IL-8 as part of innate immune system regulation (Thirkettle et al., 2013). The gene negative regulator of reactive oxygen species (*NRROS*) was highly upregulated in piglets receiving ineffective treatment. ROS are produced by phagocytes during acute bacterial infection, and their controlled release is essential for ensuring tissue recovery post-infection (Noubade et al., 2014). The observed tight regulation of ROS activity indicates that the ineffectively treated piglets maintained a balanced control of their strong innate immune response at the sampling time. Meanwhile, interleukin 1 alpha (*IL1A*) was found to also be highly expressed in ineffectively treated piglets. *IL1A*, also known as hematopoietin 1, promotes fever and sepsis during inflammation, and has numerous important functions in the inflammation process, including the stimulation of lymphocyte production (Feldmann and Saklatvala, 2001). The gene encoding complement component 5 (*C5*) displayed high expression in piglets with an ineffectively treated infection. C5, the fifth component of the complement system, plays a crucial role in both inflammatory processes and cell killing. C5 consists of an alpha and a beta chain, and the alpha chain acts as an anaphylatoxin, causing spasmogenic and chemotactic activity (Zuiderweg and Hesig, 1989).

The significantly increased expression of the selected genes (**Figure 5**; **Supplementary Data Sheet 1**) observed in this study clearly indicates that piglets receiving ineffective antimicrobial treatment with enrofloxacin had a strong immune reaction, which deviated from that of effectively treated piglets challenged with the infection. The substantially higher expression of immune genes recorded in piglets subjected to ineffective treatment underlines that these challenged piglets possess a normally functioning immune system. This expression aligns with expectations for a serious, actively developing case of colibacillosis caused by ETEC.

The analysis of immune gene expression in the two groups of piglets receiving effective and ineffective treatments clearly demonstrates the potential interplay of mechanisms behind the successful control of active ETEC infection in piglets. While using effective antibiotics is crucial, a functional immune system capable of orchestrating an array of active factors during antibiotic use is also essential.

The notable reduction in intestinal microbiota abundance that occurred after effective enrofloxacin treatment (+/− *Prob*) was expected owing to the bactericidal effect of the medicine. Conversely, sub-therapeutic enrofloxacin or ineffective tetracycline resulted in minimal changes, which was attributable to a low drug concentration and drug resistance, respectively. In mammals, including humans, it is well-established that antibiotics can change the composition of the intestinal bacteria (Grønvold et al., 2009, 2010; Lu et al., 2008). The composition of the porcine intestinal bacterial microbiota and its interaction with the host and the environment have been investigated using both culture-based and culture-independent methods (Castillo et al., 2006; Leser et al., 2002). However, studies concerning the distribution of antibiotic-resistant bacterial isolates in pig intestines are limited.

Combining probiotics with antibiotic treatments is common in veterinary practice. To mimic real-life conditions, we administered Zoolac Propaste three times daily alongside an effective dose of enrofloxacin *via* a single injection to ETEC-infected piglets. Consequently, concomitant with a marked reduction in the numbers of enrofloxacin-susceptible pathogens, the effective antimicrobial treatment likely eliminated all known probiotic microbes in the small intestine of piglets in this study. Probiotics, defined as live microbial feed supplements that benefit the host by improving its intestinal microbial balance (Fuller, 1989), promote immunity through several complex pathways, including T-cell differentiation (Casas and Dobrogosz, 2000; Ma et al., 2023). Zoolac Propaste contains four known live, five-acid-producing bacterial cultures and one thermo-stabilized bacterium. We have found that all these isolates are sensitive to tetracycline and enrofloxacin (unpublished data) employing the disk-diffusion method described by Cantas et al. (2013). Furthermore, in our previous work, we were unable to transfer the pRAS1 R-plasmid to some cultured probiotic isolates on solid surfaces (data not shown).

The ineffective antibiotic treatment group and the 0.9% NaCl control group were expected to have similar numbers of bacteria and plasmid copies. Our findings indicate that antibiotics influence plasmid and chromosomal DNA metabolism in bacteria by affecting genes that enhance the horizontal transfer of the pRAS1 plasmid and increasing the expression of the *recA* gene, thus boosting the plasticity of the bacterial genome.

The active roles of the selected R-plasmid transfer-related genes (*mobC*, *nic*, *vir*, and *tra*) in the conjugation process are well documented. *mobC* facilitates extrachromosomal gene mobility by extending DNA strand separation in the donor (Zhang and Meyer, 1997), while *nic* assists *mobC* in suppressing expression from the *mobC-nic* operon (Godziszewska et al., 2016). The *traD* gene encodes an inner membrane protein with putative ATPase activity for DNA transport during bacterial conjugation, and this protein forms a ring-shaped structure in the inner membrane through which DNA is passed to the transferosome (Kulinska et al., 2008; Lin and Kado, 1993). Furthermore, it has been shown that the *virB4* and *virD11* genes may also mediate conjugative transfer *via* a C-terminal ATPase function during pili assembly, a process that is more efficient on solid surfaces than in liquids (Porter et al., 1987; Feld et al., 2008). pRAS1 is transferred approximately 1,000 times faster on solid surfaces than in liquid media (Kruse and Sørum, 1994, unpublished data).

The expression levels of the selected genes from the conjugative transfer system varied significantly across treatment groups. The expression of transfer genes was found to be low following effective enrofloxacin treatment (+/− *Prob* or *Mx*) likely because bacteria with the pRAS1 plasmid were killed by the effective treatment, whereas treatment with a sub-inhibitory level of enrofloxacin and clinically relevant levels of tetracycline resulted in increased expression of the investigated transfer-related genes. Our results are in accordance with previous experimental data from studies on zebrafish (Cantas et al., 2013). Several factors have been proposed to explain these differences: i) the number of susceptible gut microbiota was reduced to varying degrees, thus influencing the number of potential recipients available for conjugation (Licht and Wilcks, 2006); ii) the inherent transfer potential of the donor bacteria and the genetic advantages or disadvantages conferred by the specific plasmid during conjugation with the remaining recipient population (Sandaa and Enger, 1994; Bello-López et al., 2012); and iii) the regulatory effect of the antibiotic itself on the expression levels of pRAS1 mobility genes potentially influencing transfer frequencies. Indeed, studies have demonstrated that exposure to antibiotics such as tetracycline can increase the frequency of pRAS1 conjugal transfer in sediment microcosm experiments (Licht et al., 2003).

A striking finding of the present study was the strongly increased expression levels of the selected plasmid transfer genes on the pRAS1 plasmid in the intestinal microbiota of piglets treated with ineffective antibiotics compared with the placebo group. The sub-inhibitory concentration of the quinolone enrofloxacin was chosen to mimic the low concentrations found in the intestinal lumen following intramuscular or intravenous administration for treatment purposes, in inappetent animals offered in-feed antibiotics, or through exposure to environmental residues in water. It has been shown that sub-therapeutic levels of antibiotics that normally interfere with DNA replication (e.g., quinolones) (Mesak et al., 2008; Yao and Moellering, 2003) or protein synthesis (e.g., tetracycline) (Miller et al., 2004) can induce the so-called SOS response (Salyers and Shoemaker, 1996), which is a broad bacterial DNA repair mechanism. This broad regulatory SOS response network potentially promotes the acquisition and dissemination of antibiotic resistance genes (Baharoglu et al., 2010). Thus, our results reinforce the need for great caution in the use of SOS-inducing antibiotics to avoid the induction of resistance transfer during antibiotic therapy. The observed increase in intestinal inflammation and the high prevalence of R-plasmid transfer gene activity may be connected to the impact of the host immune system on R-plasmid transfer. It has been demonstrated that inflammation can exert a promotive effect on horizontal gene transfer in the gut between a pathogenic and a commensal Enterobacteriaceae bacterium (Stecher et al., 2012).

An equally notable finding was the impact of effective antibiotic treatments on the expression levels of anti-inflammatory genes. These treatments appeared to resolve inflammation through *ALOX15* expression or the elimination of the pathogens *via* the activation of *NOX2* downstream of *SLAMF8*. Several poorly characterized genes, such as *SETD4* and *ALOX15*, were also highly expressed after effective treatment, suggesting that they play an important role in the termination of inflammation. In our study, it was not possible to investigate the differential proteogenomic impact of probiotics on the immune system owing to the poor quality of the available material after a decade of storage. Although probiotics combined with >Enr had no significant influence of on the expression level of IL-18 (0,77-fold), increased CXCL8 (13-fold) and TNF-a (5,7-fold) gene expression was found relative to that following single >Enr administration. In contrast, use of *Mx* with *>ENR* yielded 3,6-fold lower CXCL8 gene expression levels in comparison with >*Enr+Prob*. These results may indicate that the use of probiotics along with effective antibiotics may enhance the regeneration of the intestinal wall by triggering certain cytokine chain reactions. These findings offer new avenues for further research in exploring the synergy between bacterial infection treatment options with single antiobiotics or its combinations with Prob/NSAID and innate immune response. More studies are neede to discover these areas in a deeper manner.

The continuous development and growing availability of biotechnological tools have enhanced the significance and scientific value of samples collected and stored over a decade ago. We successfully obtained high-quality genetic material from almost half of the initial sample set. Notably, acquiring enough good material from the groups receiving ineffective treatment proved challenging. It is known that RNA stability is affected when acquiring samples from severely inflamed tissues because of increased Rnase activity and oxidative stress in the cells. These factors can chemically make RNA molecules less stable and more prone to degradation (Bestehorn et al., 2025). Even though the intestinal tissues were carefully and quickly immersed in RNAlater solutions after sampling, over a decade’s storage at −80°C inevitably exacerbated RNA degradation in our study.

A limitation of this study was the relatively small sample size in some of the treatment groups (*Prob*+*Mx*, n:3), which may limit the generalizability of the findings and the statistical power to detect meaningful differences in these groups. A Landrace × Yorkshire sow can have on average 14–16 piglets in each litter. While synchronized delivery was otherwise successfully achieved in this study among our four sows, unfortunately, the total number of the piglets was limited at fifty-two. Prediction of expected litter size during early gestation of our sows would have been highly beneficial for adjustments of sample size ahead in each group. Future studies can consider using transabdominal real-time ultrasonography (RTU) of the pregnant sows to predict the exact litter size and number of experimental units per treatment group (Kousenidis et al., 2022).

The use of the promiscuous R-plasmid pRAS1 in our piglet challenge model links the results from *in vitro* conjugation studies in natural microenvironments (Kruse and Sørum, 1994) to a real infection treatment model. This mammalian piglet model proved that the conjugation mechanism is independent of the bacterial environment regardless of whether the conjugation occurs in the laboratory or in the infected and diseased animal. To the best of our knowledge, this study represents the first report on how antibiotic treatment affects the expression of the bacterial SOS response and transfer genes of an R-plasmid carried by the infecting pathogen and early host signals using a piglet model.

In conclusion, tetracycline failed to control the *E. coli* infection in piglets owing to the pRAS1 R-plasmid carried by the pathogen. As expected, a similar outcome occurred when sub-inhibitory levels of enrofloxacin were employed, whereas an effective enrofloxacin dosage reduced the clinical signs and controlled both the pathogen and the transfer of pRAS1. Notably, the ineffective therapeutics—tetracycline and sub-inhibitory concentrations of enrofloxacin—increased the expression levels of plasmid mobility genes.

Millions of patients are treated with antimicrobial agents by physicians and veterinarians every day; treatment of bacterial infections is expected to become much more difficult in future owing to the emergence and spread of MDR (Aminov, 2010; Cantas et al., 2013; Podolsky, 2018). The use of subtherapeutic antimicrobials as growth promoters has been banned first in Sweden in 1986, which subsequently was followed by a complete ban in the European Union (EU) twenty years later. In accordance with our study, ineffective concentrations of antimicrobials do increase the prevalence and dissemination of resistance genes in the microbiome. This provides additional evidence and support for the prohibited use of antibiotic feed additives across the world. Despite this fact, low concentrations of antimicrobials are still used actively as feed additives in livestock for production in parts of Asia, America and China (Mulchandani et al., 2023).

Our results reveal that empiric antibiotic therapy without identification of the causative agent along with its resistance profile, and consideration of the infections that may occur in various part of the body (skin, ear, eye or intestine etc), including host characteristics, obviously accelerate the development of MDR bacterial infections. Appropriate strategies against such a challenge have already been postulated by Dr. Paul Ehrlich (Ehrlich, 1913; Williams, 2014), who for the first time addressed the art of the infection treatment at 17^th^ International Congress of Medicine over almost a century ago by suggesting ‘*Frapper Fort et Frapper Vite : Hit Hard and Hit Fast’*. Our results underline the great importance of routine bacterial identification, quick and optimal antibiotic choice according to resistance testing and correct dosage during the treatment of an individual MDR-infection to avoid further resistance development in the microbiome. In addition, we demonstrated the active role of the innate immune response, that can be enhanced by use of a suitable probiotic alongside antibiotics. Our results verify that Dr. Ehrlich's quotation remains valid even after one hundred years. Further, this study recommends following statement which partly takes its root from Dr. Ehrlich; 'Hit hard and fast, with the right antibiotic and dosage in cooperation with innate immune system of the patient’.

Numerous conjugation studies with R-plasmids indicate such a scenario; however, few plasmid transfer studies have so far been performed with a real mammalian colibacillosis model. There are good reasons to believe that the piglet model used in this study is valid for other mammals with bacterial intestinal infections that are treated with antibiotics, including humans. Our results warrant consideration by physicians and veterinarians when prescribing antibiotics, underscoring the importance of avoiding conditions that might augment the transfer of genetic drug resistance elements to commensal microbiota.

This *in vivo* study highlights the importance of a balanced and well-functioning innate immune response in the treatment of bacterial infections. The right antimicrobial choice greatly impacts the conjugative activity of an R-plasmid. These findings contribute to our understanding of the development of antibiotic resistance in infected hosts, including clinical infection treatment and control strategies. Further research is still needed to further elucidate these findings and clarify the underlying host-related mechanisms.

## Data Availability

Publicly available datasets were analyzed in this study. This data can be found here: NCBI / PRJNA1205768.
