# Supplementary material for: Impact of antibiotic choice on immune response and antibiotic resistance development in piglets experimentally infected with Escherichia coli
Source: Front Cell Infect Microbiol. 2025 Sep 9;15:1627782. doi: 10.3389/fcimb.2025.1627782 (PMC12454388; doi:10.3389/fcimb.2025.1627782)
Supplement: Supplementary file 1 [file DataSheet1.pdf]

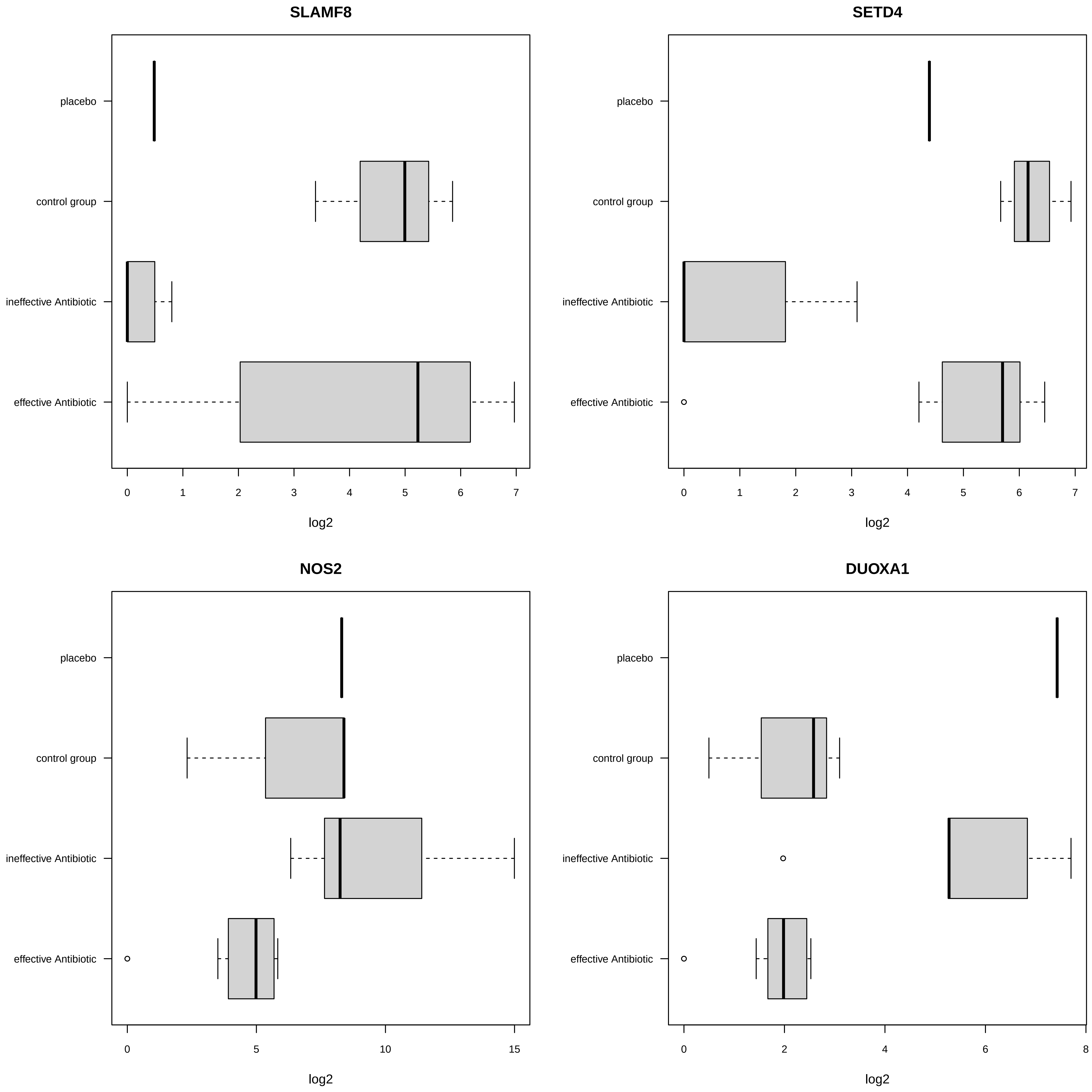
SLAMF8
placebo
control group
ineffective Antibiotic
effective Antibiotic
0 1 2 3 4 5 6 7
log2
SETD4
placebo
control group
ineffective Antibiotic
effective Antibiotic
0 1 2 3 4 5 6 7
log2
NOS2
placebo
control group
ineffective Antibiotic
effective Antibiotic
0 5 10 15
log2
DUOXA1
placebo
control group
ineffective Antibiotic
effective Antibiotic
0 2 4 6 8
log2

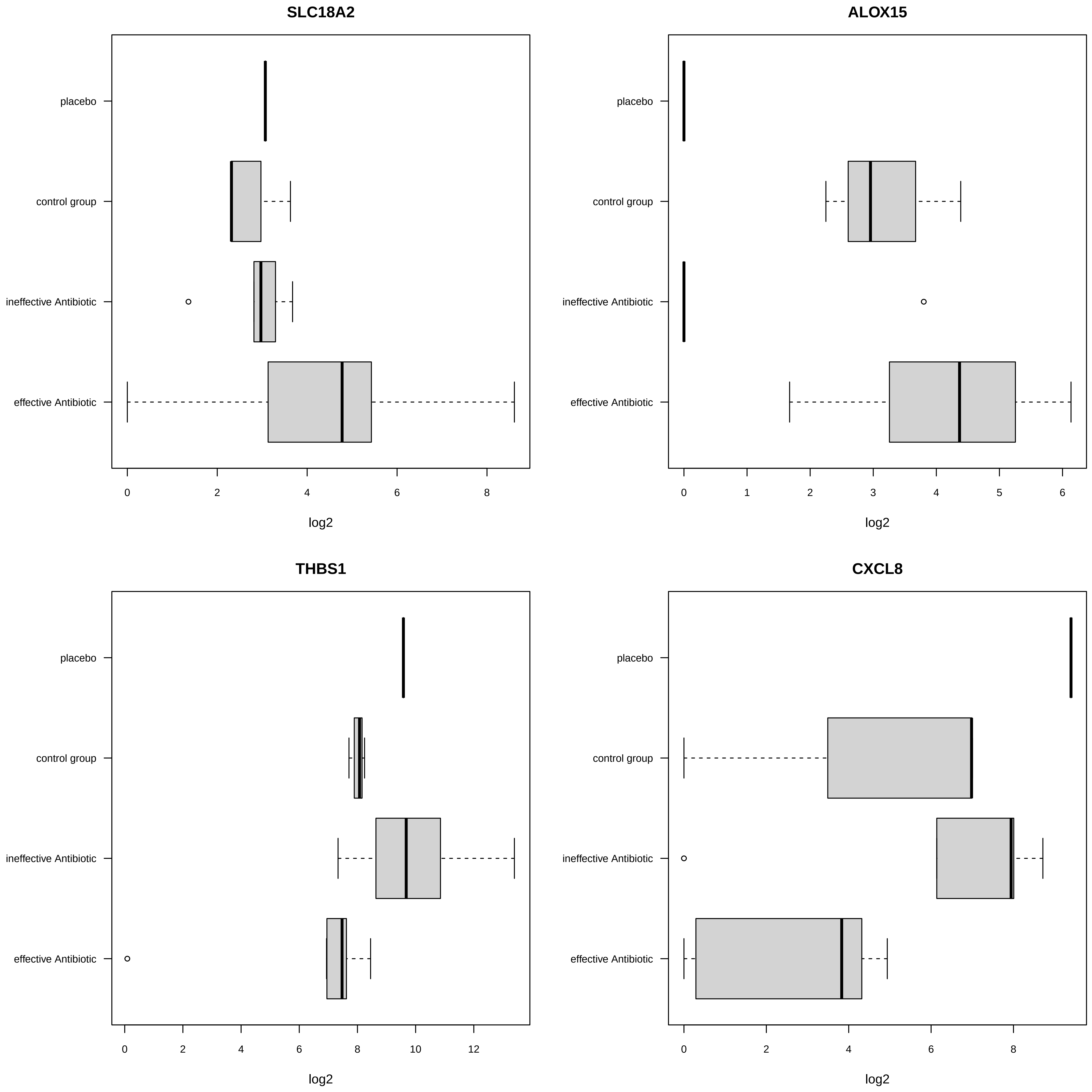
SLC18A2
ALOX15
THBS1
CXCL8
placebo
control group
ineffective Antibiotic
effective Antibiotic
log2

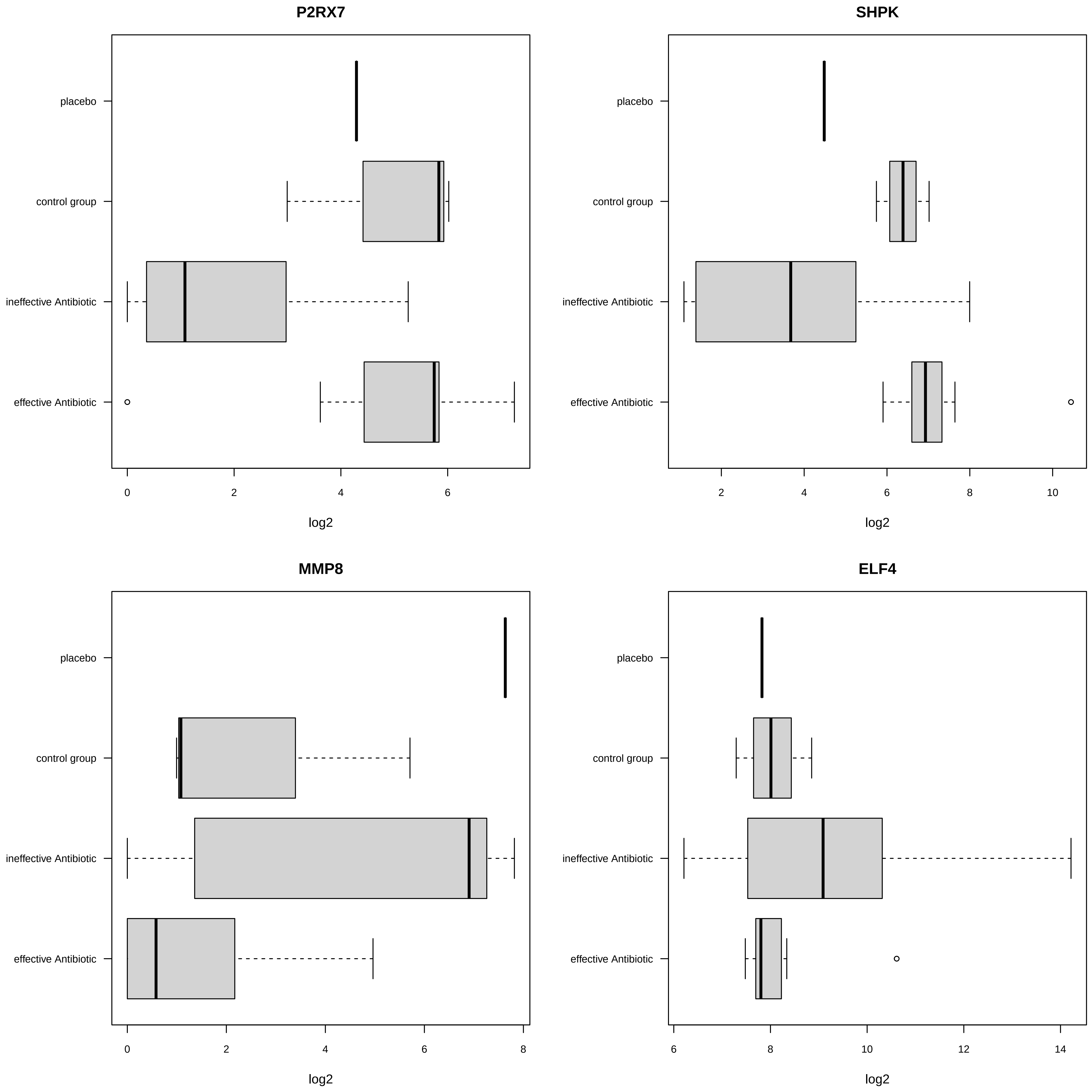

P2RX7
SHPK
MMP8
ELF4
placebo
control group
ineffective Antibiotic
effective Antibiotic
log2
0
2
4
6
8
10
12
14

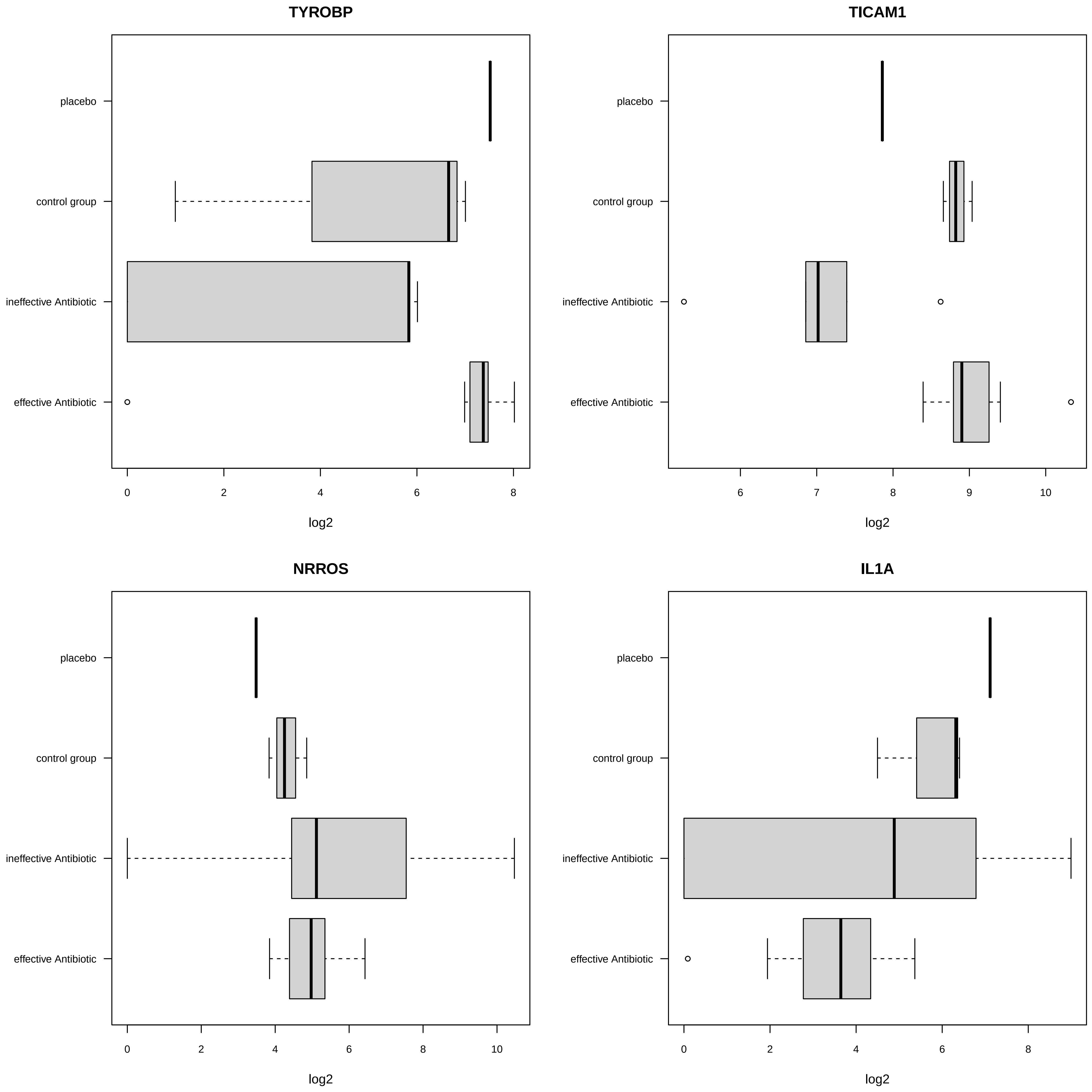

TYROBP
TICAM1
NRROS
IL1A
placebo
control group
ineffective Antibiotic
effective Antibiotic
log2
0
2
4
6
8
10

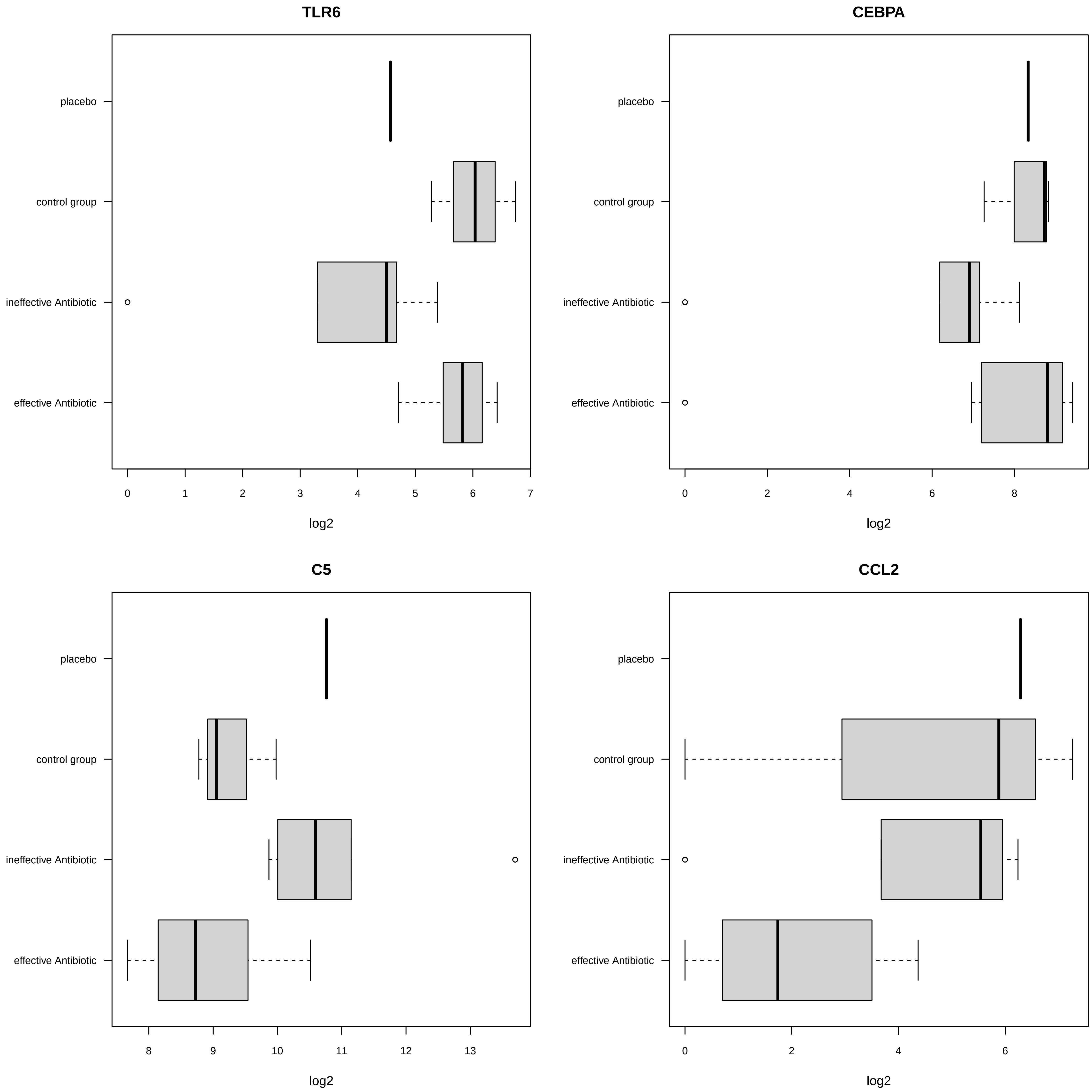
TLR6
CEBPA
C5
CCL2
placebo
control group
ineffective Antibiotic
effective Antibiotic
log2
0
1
2
3
4
5
6
7
0
2
4
6
8
8
9
10
11
12
13
0
2
4
6

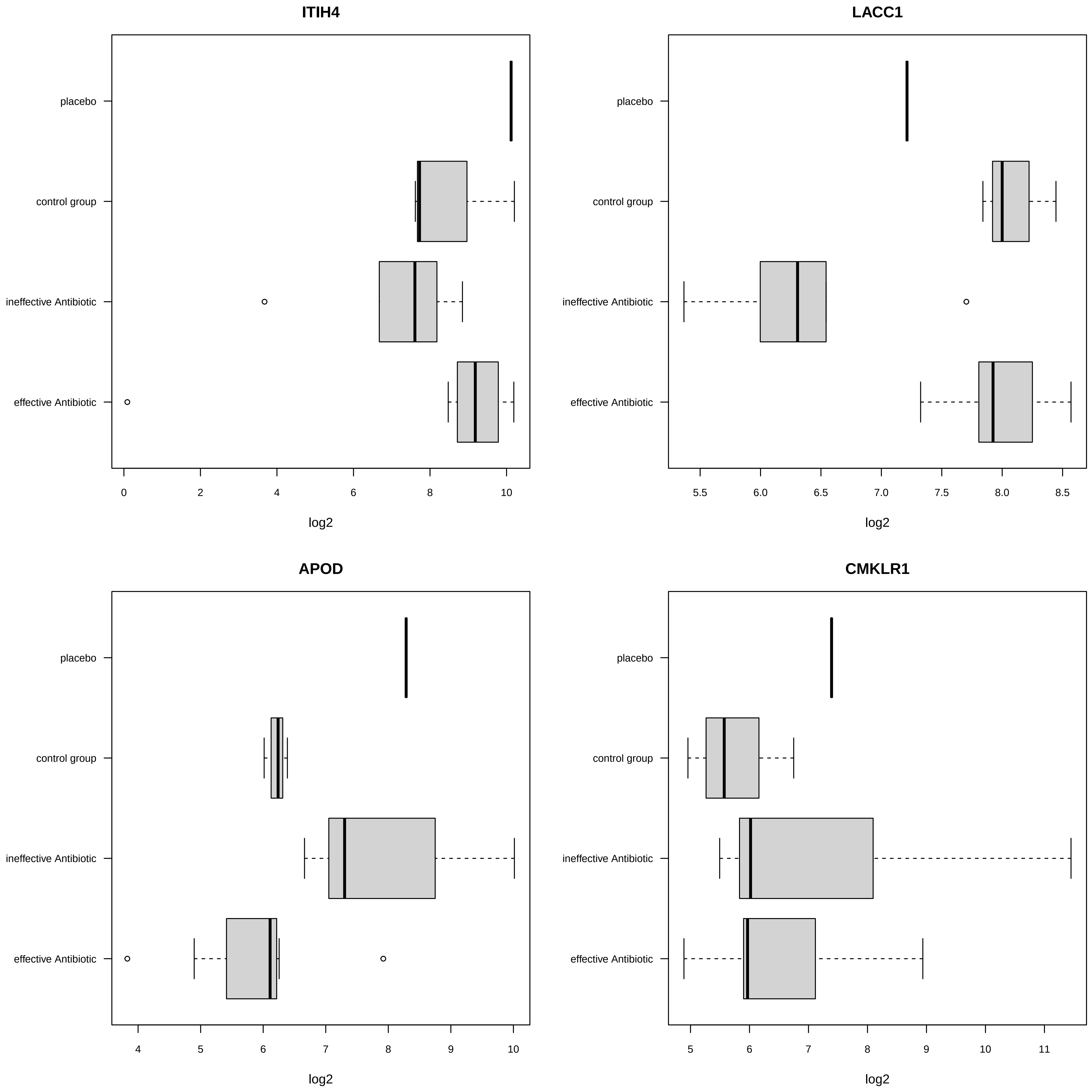
ITIH4
placebo
control group
ineffective Antibiotic
effective Antibiotic
0 2 4 6 8 10
log2
LACC1
placebo
control group
ineffective Antibiotic
effective Antibiotic
5.5 6.0 6.5 7.0 7.5 8.0 8.5
log2
APOD
placebo
control group
ineffective Antibiotic
effective Antibiotic
4 5 6 7 8 9 10
log2
CMKLR1
placebo
control group
ineffective Antibiotic
effective Antibiotic
5 6 7 8 9 10 11
log2

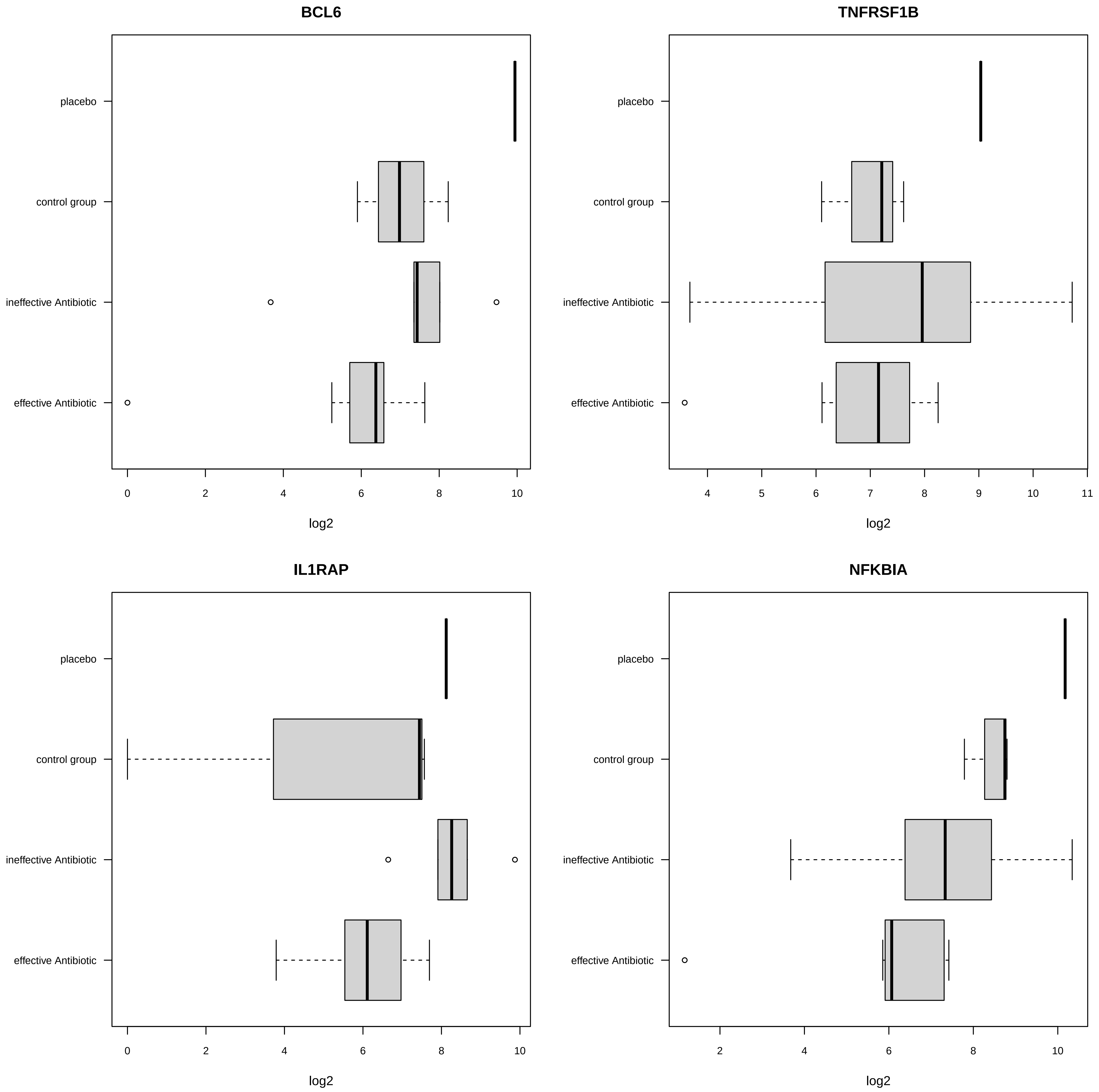
BCL6
TNFRSF1B
IL1RAP
NFKBIA
placebo
control group
ineffective Antibiotic
effective Antibiotic
log2
0
2
4
6
8
10
3
5
7
9
11
